# Properties of Spent Active Coke Particles Analysed via Comminution in Spouted Bed

**DOI:** 10.1155/2013/972985

**Published:** 2013-12-29

**Authors:** Bronislaw Buczek

**Affiliations:** AGH University of Science and Technology, Faculty of Energy and Fuels, 30-059 Cracow, Poland

## Abstract

Samples of active coke, fresh and spent after cleaning flue gases from communal waste incinerators, were investigated. The outer layers of both coke particles were separately removed by comminution in a spouted bed. The samples of both active cokes were analysed by means of densities, mercury porosimetry, and adsorption technique. Remaining cores were examined to determine the degree of consumption of coke by the sorption of hazardous emissions (SO_2_, HCl, and heavy metals) through its bed. Differences in contamination levels within the porous structure of the particles were estimated. The study demonstrated the effectiveness of commercial active coke in the cleaning of flue gases.

## 1. Introduction

Flue gases from waste incineration plants contain CO_2_, CO, steam, and NO_*x*_, as well as SO_2_, HCl, HF, and, despite the extreme conditions in which combustion is conducted, toxic organic compounds such as polychlorinated dibenzo-p-dioxins and dibenzofurans. There are also toxic heavy metals (Cd, Hg, Pb, As, and Tl) in the volatile flue gas ash. Acceptable levels of pollutants in flue gases from the combustion of municipal waste are regulated by the relevant provisions [1]. Some pollutants are removed using technology commonly applied in power stations, for example, gas dedusting with bag filters or electrostatic precipitators, desulphurisation, and NO_*x*_ removal. The reduction of dioxin emissions poses a separate problem. The methods used are divided into primary (control of the conditions of the combustion process) and secondary (reducing the concentration of dioxins in the exhaust). The most prevalent in the latter group are the flow sorption, catalytic filter, and deposit methods.

Active coke is used in the filter and deposit methods to adsorb dioxins, mercury vapour, and residues of SO_2_ and HCl. Countercurrent flow methods, for example, WKV (Warme Kraftwerkers und Verfahrenstechnik), are applied in many countries [2]. Usually the spent coke is incinerated and the bed is refilled with fresh adsorbent. It is essential to assess how much of the coke removed has been spent, but the examination of its properties per unit mass is hindered by the deposition on the surface of the residual particles of volatile ash and corrosion products from the adsorber wall.

Various physical and chemical processes have been carried out in a spouted bed. Whether or not a spouted bed is regarded as advantageous depends on the process for which it is used [3–5].

The aim of this study was to estimate the consumption of active coke by removing the external particle layers using gradual comminution in a spouted bed [6].

## 2. Materials and Methods

The commercial active coke AKP-5S produced by Gryfskand in Hajnówka (Poland) was used in final stage of cleaning flue gases from municipal waste neutralisation plant in Warsaw.

The sample of used coke was given a preliminary treatment to remove the powder produced by abrasion of the coke particles during the operation of the adsorbent bed, and the adhering vestiges of volatile powder and corrosion products were abraded from the walls of the industrial adsorber.

**Figure 1:**
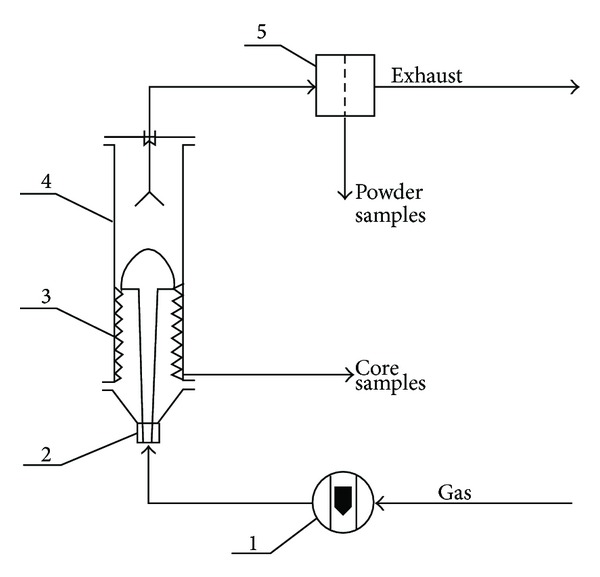
Equipment used for abrasion in a spouted bed. 1: Rotameter, 2: nozzle, 3: abrasive lining; 4: column, 5: filter.

Particles of spent and for comparison fresh active coke were subjected to comminution in a spouted bed. A diagram of the experimental equipment is shown in Figure 1.

The range of operating conditions as well as dimension of spouted bed column are given as follows:

 inert gas (N_2_) flow 0.006 m^3^/s,
 mass of active coke 0.4 kg,
 diameter of the column 0.09 m,
 height of cylindrical part of column 0.80 m,
 height of conical part of column 0.105 m,
 angle of chamber cone 45°,
 nozzle diameter 0.006 m.

**Figure 2:**
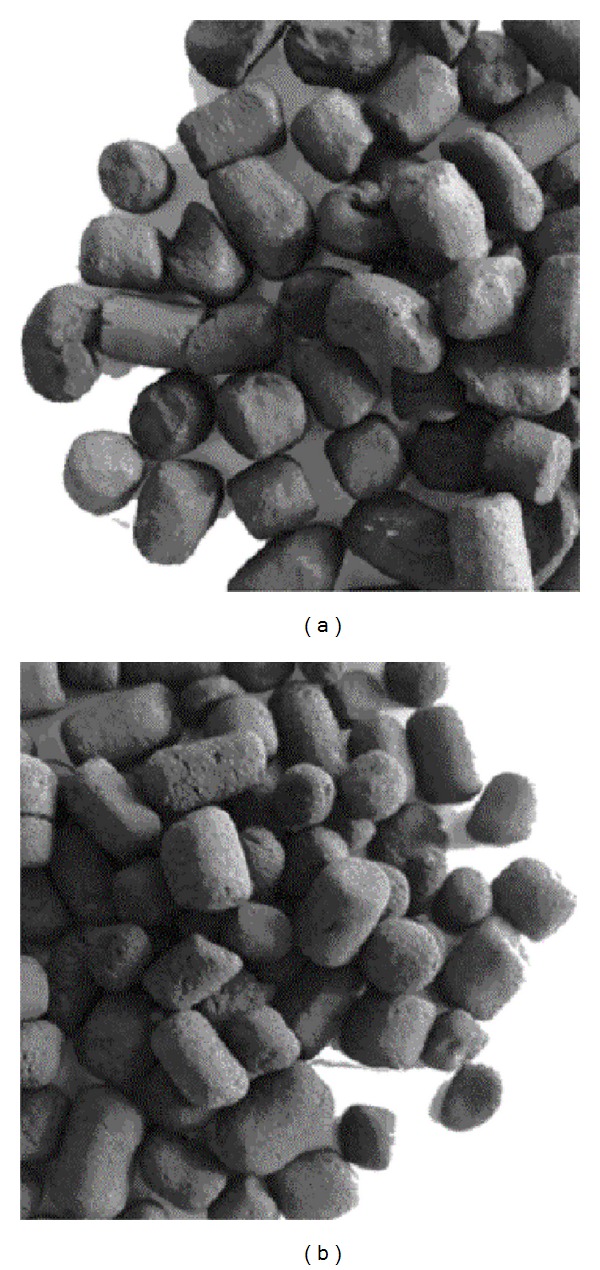
How particles of fresh active coke change in shape and dimensions after abrading.

The duration of the process was chosen in order that the amount of material abraded from the external surface of the particles in form of powder was increased to about 33 wt.%. The amount of material abraded was determined by weighing the powder and the active coke remaining in the spouted column. As a result of the attrition process, particles of fresh and spent active coke were obtained. They were designated fresh AC0, fresh AC33 and spent ACS0, spent ACS33. As a result of the preparation process in the spouted bed, particles of active cokes change in shape and dimensions. These changes for particles removed with 33 wt.% powder are shown in Figure 2.

## 3. Results and Discussion

In order to characterize tentatively the changes in the properties of the active cokes, density measurements were made and shape factors were evaluated. The results are given in Table 1.

Particles of all active cokes under investigation belong to group D according to the so-called Geldart classification [7].

The macropore volume for the cokes was obtained by mercury porosimetry [8] at 293 K using the Pascal 440 porosimeter. Mercury intrusion was applied to analyse pore radii from 5 to 7500 nm. Volumes of macropores in three radii ranges and total volume of macropores are shown in Table 2.

**Figure 3:**
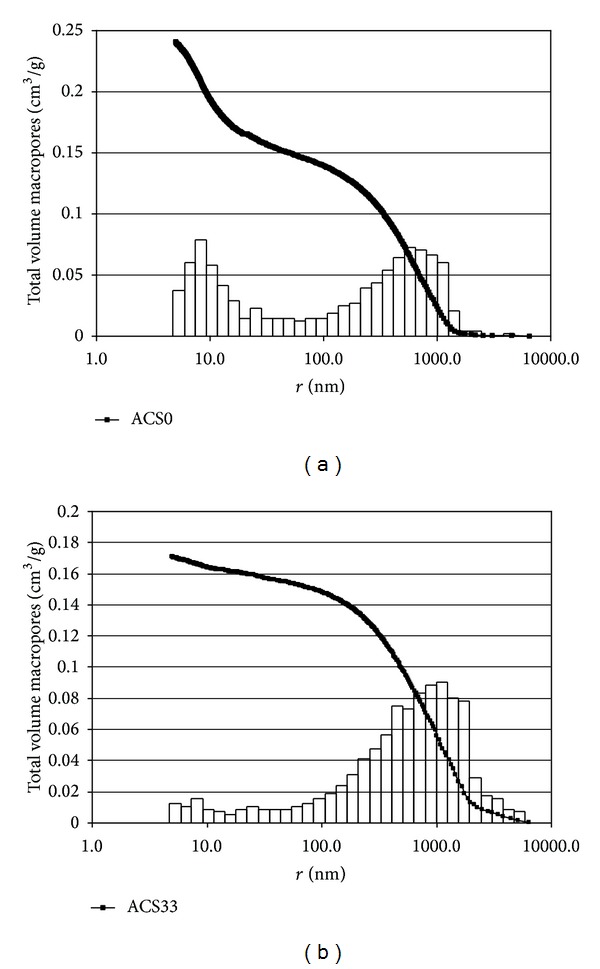
(a) Total volume macropores versus radii for ACS0 active coke. (b) Total volume macropores versus radii for ACS30 active coke.

Porosimetric investigations show a decrease in the total volume of macropores, depending on the degree of external layer removal (33 wt.%) for both active cokes. The differential dependence is observed for each macropore range. For fresh active cokes, more macropore volume is in the range radii 100–1000 nm, whereas in spent active cokes (ACS0, ACS33) maximum macropore volume was found in the ranges 100–1000 and 1000–7500 nm, respectively. This indicates that heavy metals are deposited in outer layer of particles, mainly in macropores with the largest radii. See Figures 3(a) and 3(b).

**Figure 4:**
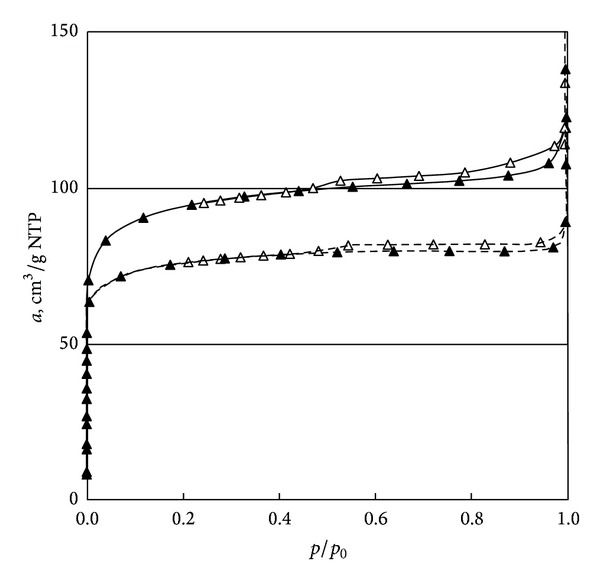
Nitrogen adsorption-desorption isotherms for spent active cokes ACS0 (solid lines) and ACS33 (dashed lines).

Texture of active cokes was analysed based on low temperature nitrogen adsorption studies. The isotherm was determined by the volume method using *a* Sorptomatic 1900 apparatus. The measurements of *a* cm^3^/g NTP were performed at the temperature of 77.5 K in the range of relative pressures *p*/*p*_0_ = 0.0001–0.999. See Figure 4.

All analysed isotherms show the Langmuir behaviour of rising adsorption in the low pressure range and a hysteresis loop appears, which proves the presence of mainly microporous and a small amount of mesoporous structures.

From the obtained data, parameters characterising the microporous structure (*W*_0_) and the characteristic energy (*E*_0_) from the Dubinin-Radushkevich equation [9] were determined. The micropore volume, the amount of adsorbed nitrogen, and the formal surface area of micropores (*S*_DR_) were calculated. The surface area of mesopores (*S*_me_) was calculated using the method proposed by Dollimore and Heal [10] and the specific surface area (*S*_BET_) was calculated by the Brunauer, Emmett and Teller equation [11]. Pore volume (*V*_*p*_) was obtained from the amount of adsorbed nitrogen under pressure of *p*/*p*_0_ = 0.98. The calculation and analysis results are summarised in Table 3.

Pore volumes for all active cokes correlate well to the results of total macroporosity obtained from mercury porosimetry.

Measurements of heavy metals were carried out on full particles (AC0, ACS0) to determine the eleven heavy metals listed in the maximum emission limits (Hg, Cd, Tl, Sb, As, Pb, Cr, Co, Cu, Mn, and Ni). These measurements were made in the Central Chemical Laboratory of Poland's National Geological Institute [12]. Mercury content was determined using a WD-XRF P7 2400 X-ray fluorescence spectrometer by Philips (The Netherlands).

Six-gram samples of the coke ground to a powder were mixed with 1.5 g of a special wax and pressed in a hydraulic press for subsequent spectroscopic determination. The remaining metals were determined in an ICP mass spectrometer, using an ELAN DRC II device by Perkin Elmer (USA). Coke samples were mineralised by microwave-supported concentrated HClO_4_, HF, and HNO_3_ acids, and the solutions obtained were examined in the spectrometer.

The values obtained in the determinations of content for the remaining heavy metals listed in provisions regulating maximum admissible concentrations for thermal waste disposal are presented in Table 4, and they show that activated coke has a fairly high efficiency for their removal.

A greater than sevenfold increase in the content of Pb, Cd, and Sb in the coke was observed, which means that the coke layer adsorbed a very substantial amount of these metals. The level of sorption for Cu, Co, and Ni was lower, only 1.2–1.4-fold. The content of Tl and As remained unchanged, while for Cr, Mn, and V there was even a slight decrease, which could be interpreted as no significant change within the bounds of experimental error. The data presented in Table 4 related to the last five metals are not necessarily evidence of the low absorptive efficiency of coke, but may be due to the absence of these metals in the volatile state in the flue gases.

## 4. Conclusion

Commercial active coke was found to be a good after-cleaning agent for flue gases generated in the incineration of municipal waste. This applies to its efficiency as a trap for SO_2_ and HCl as well as for the eleven heavy metals with limits to admissible levels of discharge designated under the relevant legal regulations. The properties of coke granules varied with respect to distance from the outer surface. It was observed that the nearer the surface, the higher the activation level, and hence the more developed the porous structure of the coke. A resulting differentiated distribution of amounts of trapped pollutants was clearly observed for SO_2_ and HCl, but not for Hg. The adsorptive properties for coke in the working temperature range of the adsorber bed and at higher temperatures may be considered stable.

## Figures and Tables

**Table 1:** Densities and shape factors (*φ* = 1/*ψ*, where *ψ* = Wadell sphericity factor).

Active coke	AC0	AC33	ACS0	ACS33
True density, kg/m^3 ^	2380	2342	2314	2243
Apparent density, kg/m^3^	1661	1416	1396	1597
Bulk density, kg/m^3^	602	587	682	585
Shape factor, *φ*	1.25–1.32	1.10–1.22	1.22–1.30	1.09–1.20

**Table 2:** Macroporosity of fresh and used active cokes.

Active coke	AC0	AC33	ACS0	ACS33
Macropores	cm^3^/g	cm^3^/g	cm^3^/g	cm^3^/g
5–100 nm	0.023	0.014	0.101	0.017
100–1000 nm	0.093	0.076	0.118	0.042
1000–7500 nm	0.055	0.031	0.022	0.124

Total macropores	0.171	0.121	0.241	0.183

**Table 3:** Analysis of pore structure and surface area of active cokes.

Active coke	*W*_0_ cm^3^·g^−1^	*E*_0_ kJ·mol^−1^	*S*_DR_ m^2^·g^−1^	*S*_me_ m^2^·g^−1^	*S*_BET_ m^2^·g^−1^	*V*_*p*_ cm^3^/g
AC0	0.115	23.2	325	11	265	0.125
AC33	0.101	24.6	385	9	235	0.113
ACS0	0.132	24.4	370	21	325	0.172
ACS33	0.112	26.8	315	12	270	0.125

**Table 4:** Contents of selected heavy metals in active coke samples.

Active coke	Content of heavy metals										
	Cd	Tl	Sb	As	Pb	Cr	Co	Cu	Mn	Ni	Hg
Admissible daily level of concentr.	0.05 mg/m^3^		∑0.05 mg/m^3^								

AC0	0.09	0.05	0.74	2.0	4.01	13	6.68	26.1	131	14.4	33
ACS0	0.69	0.05	6.36	2.0	28.8	12	8.96	31.6	129	21.1	32
